# Critical review of the *Withania somnifera* (L.) Dunal: ethnobotany, pharmacological efficacy, and commercialization significance in Africa

**DOI:** 10.1186/s42269-021-00635-6

**Published:** 2021-10-21

**Authors:** Henok Kessete Afewerky, Ayeni Emmanuel Ayodeji, Bashir Bolaji Tiamiyu, Joshua Iseoluwa Orege, Emmanuel Sunday Okeke, Aanuoluwapo Opeyemi Oyejobi, Petuel Ndip Ndip Bate, Sherif Babatunde Adeyemi

**Affiliations:** 1 Department of Neurobiology, School of Basic Medicine, Tongji Medical College, Huazhong University of Science and Technology, Wuhan, 430030 China; 2 Department of Pathology and Pathophysiology, School of Basic Medicine, Tongji Medical College, Huazhong University of Science and Technology, Wuhan, 430030 China; 3 School of Allied Health Professions, Asmara College of Health Sciences, 00291 Asmara, Eritrea; 4 Organization of African Academic Doctors, Nairobi, 00100 Kenya; 5 Department of Pharmacognosy and Drug Development, Ahmadu Bello University Zaria, PMB 1044, Kaduna, 800211 Nigeria; 6 Department of Plant Biology, Faculty of Life Sciences, University of Ilorin, Ilorin, 240001 Nigeria; 7 Department of Industrial Chemistry, Ekiti State University, PMB 5363, Ado-Ekiti, 362001 Nigeria; 8 Dalian Institute of Chemical Physics, Chinese Academy of Sciences, Dalian, 116023 China; 9 Department of Biochemistry, FBS and Natural Science Unit, SGS, University of Nigeria, Nsukka, 410001 Nigeria; 10 School of Environment and Safety Engineering, Jiangsu University, Zhenjiang, 212013 China; 11 School of Chemistry and Chemical Engineering, Huazhong University of Science and Technology, Wuhan, 430074 China; 12 Guangzhou Institute of Biomedicine and Health, Guangzhou, 510530 China; 13 C.G. Bhakta Institute of Biotechnology, Uka Tarsadia University, Bardoli-Mahuva Road, Bardoli, Surat, Gujarat 394350 India

**Keywords:** Africa, Commercialization, Ethnobotany, Ethnomedicine, Medicinal Plant, Pathophysiology, Pharmaceutics, Phytochemistry, *Withania somnifera*

## Abstract

**Background:**

*Withania somnifera* (L.) Dunal (*W. somnifera*) is a herb commonly known by its English name as Winter Cherry. Africa is indigenous to many medicinal plants and natural products. However, there is inadequate documentation of medicinal plants, including *W. somnifera*, in Africa. There is, therefore, a need for a comprehensive compilation of research outcomes of this reviewed plant as used in traditional medicine in different regions of Africa.

**Methodology:**

Scientific articles and publications were scooped and sourced from high-impact factor journals and filtered with relevant keywords on *W. somnifera*. Scientific databases, including GBIF, PubMed, NCBI, Google Scholar, Research Gate, Science Direct, SciFinder, and Web of Science, were accessed to identify the most influential articles and recent breakthroughs published on the contexts of ethnography, ethnomedicinal uses, phytochemistry, pharmacology, and commercialization of *W. somnifera*.

**Results:**

This critical review covers the *W. somnifera* ethnography, phytochemistry, and ethnomedicinal usage to demonstrate the use of the plant in Africa and elsewhere to prevent or alleviate several pathophysiological conditions, including cardiovascular, neurodegenerative, reproductive impotence, as well as other chronic diseases.

**Conclusion:**

*W. somnifera* is reportedly safe for administration in ethnomedicine as several research outcomes confirmed its safety status. The significance of commercializing this plant in Africa for drug development is herein thoroughly covered to provide the much-needed highlights towards its cultivations economic benefit to Africa.

## Background

*Withania somnifera* (L.) Dunal (*W. somnifera*) is one of the most popular medicinal plants of great importance, used in Africa and the world at large. It is among the 85 most prominent African medicinal plant species of international trade per surge in research publications of over 1767 within the last decade (VanWyk 2015). This plant is a member of Solanaceae, a family with 84 genera and about 3000 species that are diversely distributed throughout the world (Mirjalili et al. 2009). *Withania* is a genus with species that are shrubs, sub-shrubs, or woody herbs. *W. somnifera* is an ecologically and economically important plant that is as old as Ayurvedic medicine, with millennia of usage as one of the essential plants used in Ayurvedic medicine, the Mediterranean region, and Orientalis. It is a widely distributed plant mainly in Africa, Asia, Australia, and Europe. In India, it is readily available via cultivation and in the wild agricultural land; and aside from being medicinal, it is also used as bioremediation for phytoextractions (Burkill 1985; Abhilash et al. 2008; Singh and Kumar 2011).

In Africa, *W. somnifera* exists in several countries, including South Africa, Lesotho, Sudan, South Sudan, Djibouti, Egypt, Tanzania, Swaziland, Mali, Nigeria, Liberia, and Congo (Burkill 1985; Iwu 2014; Naveen et al. 2015). *W. somnifera* is reported to be indigenous to South Africa, where it is commonly used as a sedative and hypnotic, and also regarded to be effective against numerous ailments in southern Africa, including Lesotho (Burkill 1985; Dold and Cocks 2000). Different parts of the plant are used for various purposes; for example, ointment from leaves and berries is applied to treat cuts, wounds, abscesses, and inflammation. The leaf decoction is also employed to treat haemorrhoids and rheumatism (Idowu and Wilfred 2018). The *W. somnifera* root contains over 35 bioactive molecules that account for its multi-properties. The plant root is used in traditional medicine as a popular supplement, with supposed benefits that include lessening anxiety and stress (Vijay et al. 2018). In addition to its high antioxidants content, the consumption of the roots improves cardiovascular health, reduces swelling and stress, strengthens heart muscles, regulates cholesterol, and reduces hair loss in the human body (Mamta and Vijaya 2018). Moreover, the roots have also been used for veterinary purposes to treat cattle. A decoction of cooked roots and leaves fed to sheep, cows, and buffalo enhances milk production, while it is also used as an antipyretic and sexual tonic (Aziz et al. 2018a). It is also a rich source of both micro- and macro-nutrients, including iron, magnesium, phosphorous, copper, zinc (Sangita and Alka 2016). Additionally, *W. somnifera* root extracts are common trade goods in the cosmetics and personal care industries, such as skin conditioners, shampoos, and antiwrinkle agents (Sakamoto et al. 2017). In Egypt, Djibouti, and Ethiopia, people widely use the plant to treat Alzheimer’s disease, bronchitis, and malaria (in combination with other plants) (Hassan-Abdallah et al. 2013; Alebie et al. 2017; Rahma et al. 2017).

*W. somnifera* is one of the most essential ethnomedicinal herbs in Ayurveda medicine due to its wide range of therapeutic actions (Singh et al. 2011). The ethnomedicinal usages of *W. somnifera* (Table 1) suggest the significance of the plant for further pharmacological scientific research against several pathophysiological conditions. Noteworthy, no mutagenicity and genotoxicity have been reported for *W. somnifera*, and thus, the plant has been cleared as a safe use for the management of neurocognitive disorders, diabetes, arthritis, and a lot of other disease conditions (Singh et al. 2011). However, some mild and brief-type adverse events such as epigastric discomfort and loose stools were reported as most common, whereas drowsiness, hallucinogenic, nasal congestion (rhinitis), cough, cold, decreased appetite, nausea, constipation, dry mouth, hyperactivity, nocturnal cramps, blurring of vision, hyperacidity, skin rashes, and weight gain were reported as less common adverse side effects (Tandon and Yadav 2020).

**Table 1:** Ethnomedicinal usages of *W. somnifera*

Ethnomedicinal usage	Plant part	Geographical location	References
Nutrition	Young shoots	South Africa, India	Duke et al. (1985)
Eye treatments	Leaf	Kenya	Duke et al. (1985)
Abortifacients	Leaf, root	Morocco (used with other plants), South Africa	Merzouki et al. (2000), Moroole et al. (2019)
Sedatives	Root	South Africa	Elizabeth and Dabur (2002), Umadevi et al. (2012)
Aphrodisiac	Flower and dried mature roots	Tanzania, India	James (2002), Chauhan et al. (2014)
Kidneys, Fever	Leaf, root	South Africa	Van et al. (2008)
Skin infection (Smallpox, Measles, etc.)	Leaf, stem, and berry	South Africa, Jordan	Al-Qura'n (2009), Mabona and Van (2013)
Ethnoveterinary (Anthrax)	Dried roots	Ethiopia	Eshetu et al. (2015)
Gynaecological	Leaf, fruit, root	Lesotho, Pakistan	Moteetee and Seleteng (2016), Aziz et al. (2018b)
Haemorrhoids	Green fruit	Iran	Hashempur et al. (2017)
Pulmonary troubles	Leaf, root	Pakistan	Alamgeer et al. (2018)
Asthma and Malaria	Leaf-sap	Pakistan	Umair et al. (2019)

The raises in *W. somnifera* market demands in Africa and elsewhere mostly associate with its extensive medicinal, nutritional, and cosmetic applications. In particular, its uses in the productions of pharmaceutical drugs and products for human consumption, including energy drinks, capsules, health supplements, energy boosters, and food product materials, have been remarkably notable (Jayanta et al. 2018). Sales in these forms are flourishing worldwide due to its reputation as a natural solution to health, beauty, and nutrition-related problems. The vast majority of *W. somnifera* in the market is supplied to herbal products and dietary supplement manufacturers in the form of dry extract, with a global market value worth over $12 million (Vineet et al. 2018). In Africa, the sale of *W. somnifera* is exercised in open markets as well as in herbal shops. For example, in the Eastern Cape Province, herbal cosmetic products are more frequently bought from herbal shops, but in a few cases, they are also still prepared at home, especially those used for skincare management (Idowu and Wilfred 2018). The major companies involved in the *W. somnifera* extract market include Life Extension, Taos Herb Company, General Nutrition Centers, Jarrow Formulas, Hugh Mountains, Organic India, and Vitamin Shoppe. The main market types include capsule and liquid, while the main applications include herbal products and drugs (Jon 2017). The USA is the largest *W. somnifera* market, with over 40% of products sold at the end of 2017 contained this plant. These products mainly included dietary supplements, sports nutrition products, and functional food (beverages, bars, and snacks). The main reason for the broad market of the plant in the USA is likely due to its constituent’s antistress, antifatigue, and anti-insomnia properties, among others. Other countries with profitable markets include the UK, India, and Germany (Jon 2017).

## Review methodology

This review focuses on recent studies that underscore the *W. somnifera* degrees of use, highlighting its ethnography, ethnomedicinal usage, phytochemistry, and toxicological effects. The potential pharmacological efficacy of *W. somnifera* against several pathophysiological conditions is herein thoroughly discussed. Additionally, the significance of commercializing the plant in Africa for drug development is also extensively reviewed to provide the much-needed highlights towards its benefit to Africa. Scientific articles and publications were scooped and sourced from high-impact factor journals and filtered with relevant keywords on *W. somnifera*. Several scientific databases, including GBIF, PubMed, NCBI, Google Scholar, Research Gate, Science Direct, SciFinder, and Web of Science, were accessed for surveying the most influential articles and recent breakthroughs published in the context of *W. somnifera*.

## Ethnography and ethnomedicinal usage of *W. somnifera*

### Ethnography of *W. somnifera*

**Fig. 1:**
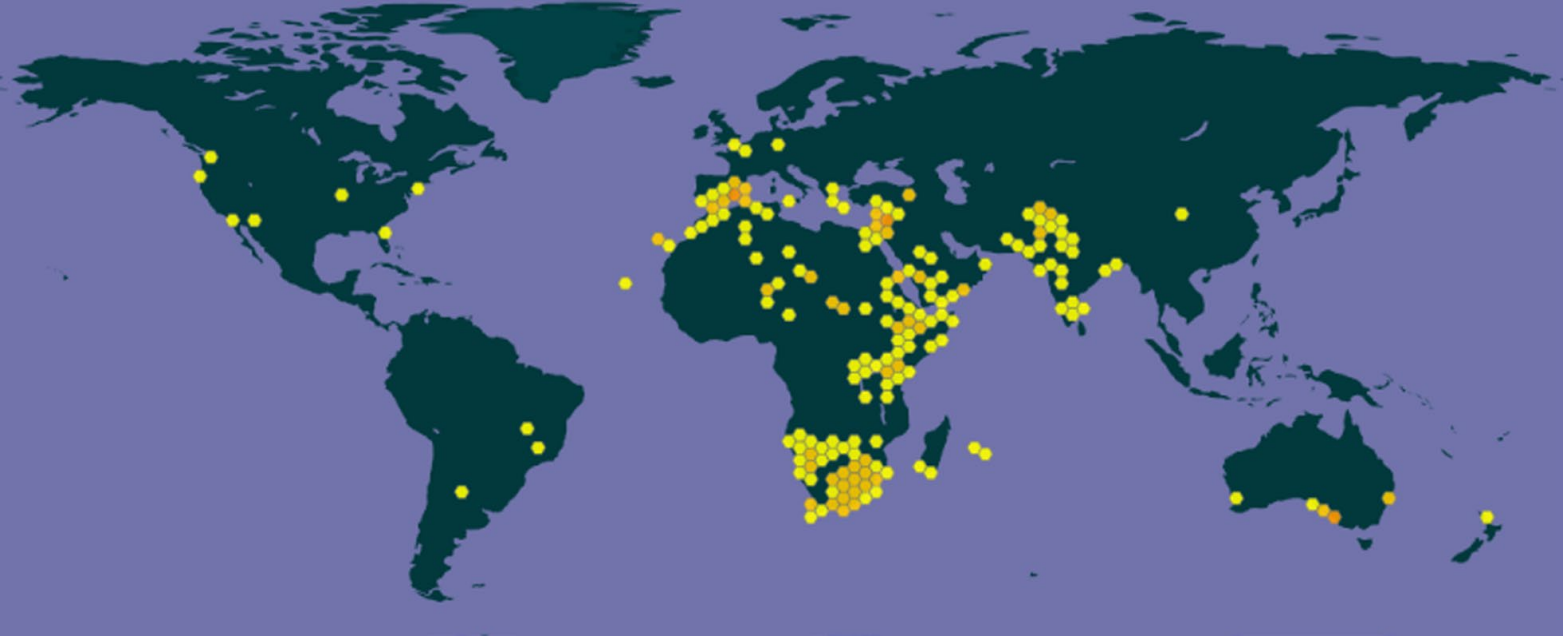
Ethnographic distribution of *W. somnifera* (*Withania somnifera* (L.) Dunal in GBIF Secretariat 2021)

*W. somnifera* is commonly known as Tarkukai in the Marakwet community of Kenya, Karamanta in the northern part of Nigeria, Zafua in Mali, and Winter Cherry in South Africa (Burkill 1985). The distribution area of *W. somnifera* extends from the Canary Islands and the Mediterranean region through Africa, the Middle East, India, and Sri Lanka to China (Schmelzer et al. 2008). It possesses a natural occurrence, most abundantly in dry and humid regions (Gaurav et al. 2015). In Africa, *W. somnifera* is present in the northern-, southern-, and eastern-region countries and few countries in the western-region (Gaurav et al. 2015). In the north African countries, *W. somnifera* is common in Morocco, Algeria, Tunisia, Libya, Egypt, and Sudan but absent in Western Sahara. The plant is present in all countries in the southern part of the African continent. Its presence has also been recorded in Chad, Cape Verde, Mali, Liberia, and Nigeria in the western part of Africa. In the eastern part of the continent, countries including Ethiopia, Tanzania, Angola, Zambia, Mozambique, and Eritrea have this plant growing there (Fig. 1) (*Withania somnifera* (L.) Dunal in GBIF Secretariat 2021).

### Ethnomedicinal usage of *W. somnifera*

**Fig. 2:**
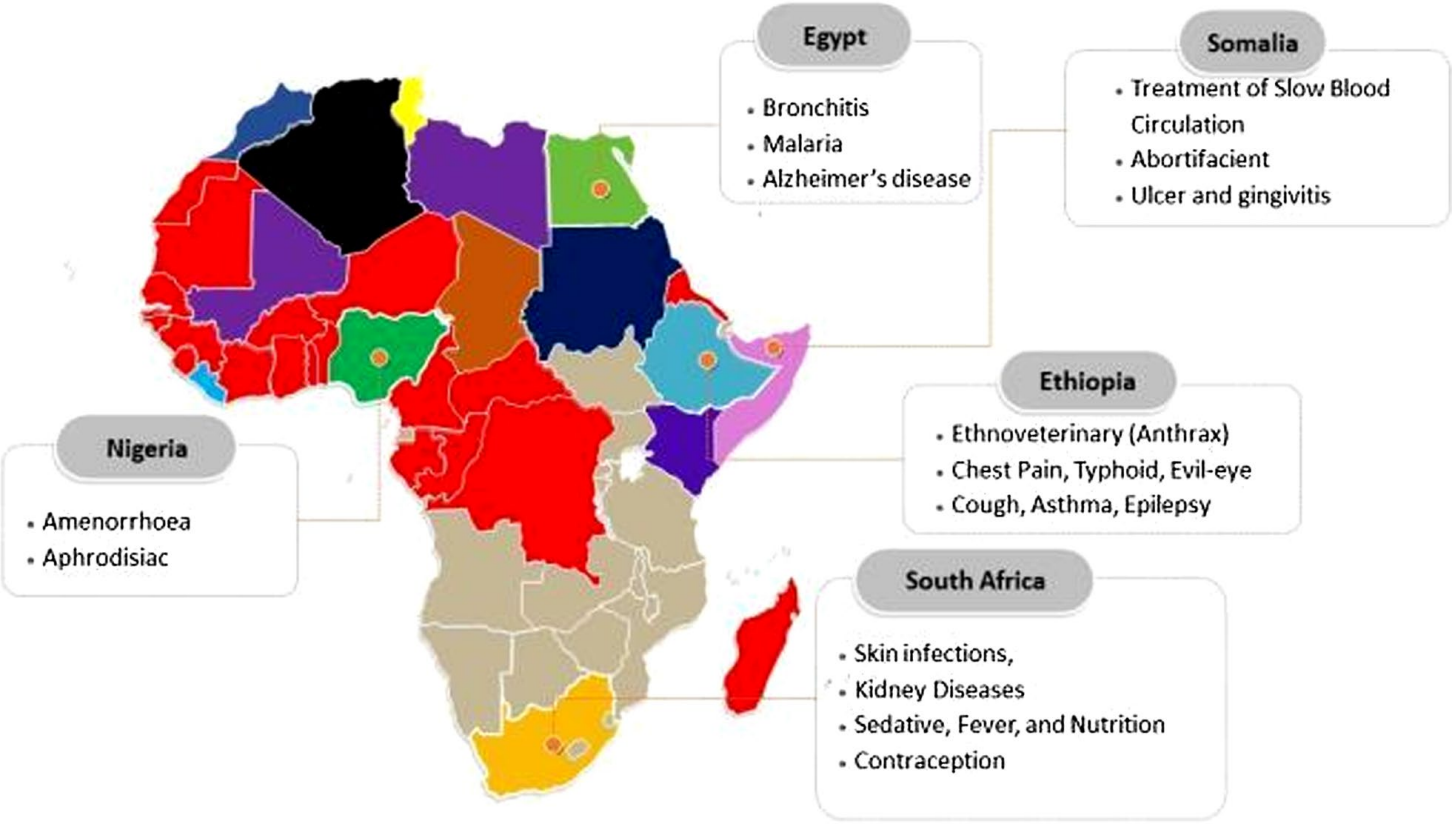
Ethnomedicinal usage distribution of *W. somnifera* in African countries and the plant applications in selected countries

Extensive usage of *W. somnifera* in Africa for medicinal purposes (Fig. 2) has been documented in literature (Burkill 1985; Schmelzer et al. 2008; Moroole et al. 2019). Among the 23 species present in the *Withania* genus, *W. somnifera* is economically more valuable because of its medicinal properties (Kulkarni and Dhir 2008; Mirjalili et al. 2009). In Cape Verde, locals commonly use an infusion of the *W. somnifera* leaves for blood purification and the whole plant as a diuretic and antibacterial, such as against gonorrhoea (Schmelzer et al. 2008). Notably, the people of Ethiopia are extensively reported to frequently make use of *W. somnifera* to treat cough, asthma, epilepsy, eye infections, scabies, and paralysis, as well as evil eyes and evil spirit (Giday et al. 2003; Wondimu et al. 2007; Teklay et al. 2013). Moreover, the Zay people of Ethiopia are exclusively documented to also employ *W. somnifera* in the treatments of chest pain and typhoid (Giday et al. 2003).

The smoke of a burning *W. somnifera* is wafted over patients with slow blood circulation in Somalia (Raut et al. 2012). In Sindhi, the leaves or roots are pounded together with the parts of other plants and administered as an abortifacient (Saiyed et al. 2016). The *W. somnifera* leaves are purgative, and therefore, its crushed forms are used as a general body pain reliever, including in the treatment of chronic skin ulcers (Kipkore et al. 2014). *W. somnifera* is employed for contraception and other reproductive problems in the Eastern Cape, Free State, and Kwazulu-Natal of South Africa (Moroole et al. 2019). There is reported usage of *W. somnifera* in combination with leaves of *Ensete vetricosum*, whereby both are boiled together to treat candida infection in Venda, South Africa (Masevhe et al. 2015). Also, the leaves of *W. somnifera* are used in wound healing by applying on open wound abscesses by the people of the Eastern Cape of South Africa (Grierson and Afolayan 1999). In the Lagos state of Nigeria, people commonly use *W. somnifera* for amenorrhoea and aphrodisiac purposes (Sharaibi et al. 2017). Given the vast ethnomedicinal usage, the findings of numerous recent studies in clinical and experimental settings further reveal the efficacy of *W. somnifera* for the management of several pathological conditions and provide added substantial highlights on its medicinal significance (Priyanka et al. 2020; Fuladi et al. 2021).

## Phytochemistry and toxicological studies of *W. somnifera*

### Phytochemistry of *W. somnifera*

Through the application of various chromatographic and spectroscopic techniques, the phytochemical constituents of *W. somnifera* root, leaves, stem, and fruit extract have been extensively explored and characterized (Mirjalili et al. 2009). These phytochemical investigations on different parts of *W. somnifera* plant extracts indicate the presence of several biochemically active components, including alkaloids, phenols, flavonoids, saponins, tannins, carbohydrates, steroidal lactones, β-sitosterol, scopoletin, sitoindosides, somniferiene, somniferinine, pseudotropine, anaferine, anahygrine, cysteine, chlorogenic acid, cuscohygrine, withanine, withanolides, withananine, tropanol, 6,7β-Epoxywithanon and 14-α-hydroxywithanone (Naz and Choudhary 2003; Saleem et al. 2020). Noteworthy, these extract constituents are held responsible for the various biological activities of effect in the plant ethnomedicinal usage and pharmacological efficacy (Aye et al. 2019; Nile et al. 2019). Specifically, the *W. somnifera* whole plant and its different parts phytochemical extracts composition outline as indicated below: -

1. The *W. somnifera* whole plant extract is rich in phytochemicals, such as alcoholic extract of the plant contains anaferine, anahygrine, choline, cuscohygrine, pseudotropine, *dl*-isopelletierine, and tropine (Saleem et al. 2020). Besides, the methanolic extracts of the plant constitute starch, acylsteryl glucosides, iron, ducitol, hantreacotane, withaniol, and amino acids such as alanine, aspartic acid, cysteine, tyrosine, glutamic acid, glycine, proline, and tryptophan (Mirjalili et al. 2009; Alam et al. 2011). The aqueous extract of the whole plant contains withanone and tubacapsenolide F (Saleem et al. 2020), while similar extracts with equimolar ratios of water and methanol constitute chlorinated withanolide and 6α-chloro-5β,17α-dihydroxywithaferin A along with nine withanolides, namely 6α-chloro-5β-hydroxywithaferin A, (22R)-5β-formyl-6β,27-dihydroxy-1-oxo-4-norwith-24-enolide, 2,3-dihydrowithaferin A, withanone, withanoside IV, withaferin A, 2,3-didehydrosomnifericin, 3-methoxy-2,3-dihydrowithaferin A, and withanoside X (Saleem et al. 2020). The ethanol extract of the whole plant constitutes isosominolide, sominone, withasomniferin A (Misra et al. 2008).

2. Extract of *W. somnifera* root using alcohol extractant yields a pyrazole alkaloid, withanolide A, and withasomnine (Baek et al. 2019). Methanolic extract of the plant root constitutes withanosides I-VII (Menssen and Stapel 1973; Mirjalili et al. 2009), whereas three withanolides, namely withanolide A, B, and C, make the benzene and ethyl acetate extracts of similar roots (Kim et al. 2019). β-sitosterol and *d*-glycoside are the major phytochemical components of the plant’s root extraction with petroleum ether and acetone (Saleem et al. 2020). Butanol extracts of *W. somnifera* roots display the phytochemical existence of physagulin, withanoside IV, and withanoside VI (Mathur et al. 2006; Chatterjee et al. 2010).

3. Leaves of the plant extracted with methanol show the phytochemical presence of ashwagandhine, cuscohygrine, *dl*-isopelletierine, somniferine, tisopelletierine, 3α-tigloyloxtropine, 3-tropyltigloate, hygrine, hentriacontane, mesoanaferine, visamine, withanine, withananine, withasomnine, and pseudowithanine (Siddique et al. 2014). Alcoholic extract of the plant leaves constitutes withanolide D, E (Lavie et al. 1968, 1972), withanolides F–M (Glotter et al. 1973, 1977), withanolides N, O (Ganzera et al. 2003), and withanolide P (Glotter et al. 1977). Leaves of the plant extracted with ethanol contain (5R,6S,7S,8S,9S,10R,13S,14S,17S,20R,22R)-6,7α-epoxy-5,17-α,27-trihydroxy-1-oxo-22R-witha-2,24-dienolide (Ben et al. 2018). Methanolic extract of the plant roots constitutes numerous dragendorff positive alkaloids that are phytochemicals recognized as anaferine, anahygrine, choline, pseudotropine, cuscohygrine, isopelletierine, *dl*-isopelletierine-3-tropyltigloate, hentriacontane, hygrine, mesoanaferine, somniferine, 3α-tigloyloxtropine, visamine, withanine, withananine, and withasomnine along with ashwagandhine, pyrazole derivatives, and pseudowithanine (Saleem et al. 2020).

4. Ethanolic extract of the *W. somnifera* stem bark mainly contains withanolides, including somniferanolide, somniwithanolide, somniferawithanolide, withasomnilide, and withasomniferanolide (Siriwardane et al. 2013; Saleem et al. 2020).

5. Oil extracts from fresh berries of *W. somnifera* contain saturated and unsaturated fatty acids such as elaidic acid, linoleic acid, oleic acid, palmitic acid, and tetracosanoic acid (Sidhu et al. 2011). Methanolic extract of the plant fruits possesses withanamides A-I (Bhatia et al. 2013) and 6,7α-epoxy-1α,3β,5α-trihydroxy-witha-24-enolide (Adhikari et al. 2020).

### Toxicological studies of *W. somnifera*

Studies have revealed the safety profile of several extracts of *W. somnifera* for all age groups and sexes, even during pregnancy (Raut et al. 2012; Patel et al. 2016). Administration of 2000 mg/kg of body weight of the hydroalcoholic extract for acute and sub-acute oral toxicities in albino rats of Wistar strain is determined to be practically safe (Patel et al. 2016). Other reported studies also indicate that 1260 mg/kg of bodyweight mark the mean lethal dose (LD_50_) of this plant extract in Swiss albino mice, and any increase above this dose limit is proven to lead to the death of the treated mice (Sharada et al. 2008; Dar et al. 2015). In a similar study, the administration of *W. somnifera* extract in Wistar rats showed no toxicologically significant treatment-related changes in biochemical observations, ophthalmic examination, bodyweight changes, feed consumption, as well as organ weight, even at 2000 mg/kg of bodyweight high dose (Prabu et al. 2013).

Evaluations of dose-related tolerability, safety, and activity of *W. somnifera* formulation (aqueous extract in capsules ranging from 750 to 1250 mg/day) in normal individuals are experimented with (Raut et al. 2012). Finding on the formulation are reported safe and shown to strengthen muscle activity. A sub-acute toxicity study involving a combination of *W. somnifera* and *Panax ginseng* also revealed no significant toxic effect on studied parameters (Aphale et al. 1998), hence, substantiating the claim of *W. somnifera* safety for consumption. A recent survey of studies that involved several preclinical scientific studies and clinical trials on the toxicity of *W. somnifera* establishes evidence on its extracts (aqueous, ethanolic, or hydroalcoholic) reasonable safety for herbal medicine use (Tandon and Yadav 2020).

## Potential pharmacological efficacy of *W. somnifera* against pathophysiological conditions

### Cardiovascular diseases

*W. somnifera* is widely used as a therapeutic drug in Ayurveda and Unani medicines (Palliyaguru et al. 2016). This plant is known to grow in Asia, Africa, and the Mediterranean region and has been extensively used in African Traditional Medicine (ATM) for managing different pathological conditions, including cardiovascular diseases (Dar et al. 2015; Chukwuma et al. 2019).

Cardiac-related infarction is one of the major leading causes of death globally (Siegel et al. 2016). Interestingly, the findings of reported studies indicate the *W. somnifera* therapeutic significance in ameliorating myocardial infarction (Bilal et al. 2012). The antioxidant activity and the antiapoptotic properties of the *W. somnifera* extracts are confirmed to have a significant cardioprotection effect based on the myocardial and antioxidant histopathological evaluations (Mohanty et al. 2004, 2008). Another study reported on the *W. somnifera* extracts potential cardio-tonic and cardioprotective effects in preventing myocardial infarction and ischaemia–reperfusion injury to the heart also proven the therapeutic value of the herb extracts in the cardiovascular context (Raghavan and Shah 2015). Further, an in vivo study on the biochemical and histopathological parameters showed that the extract of *W. somnifera* protects the myocardial cell membrane due to its antilipoperoxidation and antioxidants effects (Khalil et al. 2015). The acute toxicity of the *W. somnifera* extract at 2000 mg/kg is determined practically safe, and its administration presents low toxicity (Prabu et al. 2013; Patel et al. 2016), however significant to combat many pathophysiological diseases.

Reported studies indicate that root extracts of *W. somnifera* enhance cardiorespiratory endurance and improve the quality of life among healthy athletic adults, while suggesting a careful selection of doses to prevent heart failures (Sandhu et al. 2010; Choudhary et al. 2015; Perez-Gomez et al. 2020). An Auto-Dock-Vina model on different proteins associated with cardiovascular diseases enlightens that Withaferin A is a potential lead compound that can inhibit cardiovascular diseases (Ravindran et al. 2015).

Furthermore, reported study findings suggests that Withaferin A inhibits apoptosis via activated oxidative stress demonstrating mechanisms of action in the H_2_O_2_-induced oxidative stress injury model through improved cell survival and reduced oxidative stress, which all insight to this *W. somnifera* bioactive constituent utilization as a drug candidate for the treatment of cardio-related diseases (Yan et al. 2018b). The root powder of *W. somnifera* at 50 mg/kg of body weight (BW) and 100 mg/kg BW significantly reduces right-ventricular pressure and other parameters on mono-crotaline induced pH in rats by remarkably improving in inflammation, oxidative stress, endothelial dysfunction, attenuation of proliferative markers, and apoptotic resistance in lungs (Kaur et al. 2015).

Interestingly, findings of a research study on the therapeutic efficacy of the root powder of *W. somnifera* for the management of hypertension suggest that the intake of the root powder with milk decreases systolic blood pressure (Kushwaha et al. 2012). Further, in a meta-analysis review on the active chemical compounds of *W. somnifera*, Withaferin A is underlined to have therapeutic potential against COVID-19 pandemic infection-induced cardiorespiratory disease (Straughn and Kakar 2020). The study suggests that the Withaferin A steroidal lactone ring could mitigate the virus-induced cardiovascular pathological features (Afewerky 2020) through the mechanisms that include anti-inflammatory actions and even binding to the viral spike (S-) protein of SARS-CoV-2.

### Neurodegenerative diseases

Mainly over the last couple of decades, with the increasing life expectancy, the incidence and prevalence of neurodegenerative diseases such as Alzheimer’s, Parkinson’s, and Huntington’s diseases have increased substantially (Lopez and Kuller 2019). Therefore, the public health impact of neurodegenerative diseases in the ageing baby boom generation requires priority attention. To address this concern, several preliminary research, preclinical studies, and clinical trials have been undergoing. However, because of the disease’s pathophysiological complexity, there is no efficient pharmaceutical approach for the prevention or treatment of neurodegenerative diseases at the moment.

While further research is required, the ongoing pharmaceutical investigation efforts for the therapeutics of neurodegenerative diseases signify the potential role of medicinal plants, such as *W. somnifera*, extracts than the use of synthetic approaches in terms of both treatment efficacy and access (Pohl and Kong 2018). *W. somnifera* has a long history as a medicinal plant to treat various neurodegenerative conditions (VenMurthy et al. 2010). Neurodegenerative diseases are characterized by a slow and progressive deterioration of the central nervous system structure and function (Dugger and Dickson 2017). Mainly over the last decade, *W. somnifera* has gained extra attention in the context of treatment for neurodegenerative ailments, including loss of memory (Uddin et al. 2019), bipolar disorder (Chengappa et al. 2013), and locomotor defects (Manjunath and Muralidhara 2015). Interestingly, the most reported neuroprotective mechanisms of *W. somnifera* extracts against several neurodegenerative diseases include the restoration of mitochondrial function concurrent with the mitigations of oxidative stress, inflammation, and apoptosis (Dar et al. 2017; Birla et al. 2019; Gupta and Kaur 2019).

#### Role of *W. somnifera* against Alzheimer’s diseases

Alzheimer’s disease (AD) is the most common form of neurodegenerative disease that causes progressive impairment of higher cognitive function, memory, and social skills (SoriaLopez et al. 2019). The pathogenesis of AD is not yet fully elucidated. However, age is considered as the AD primary risk factor, and the aggregation of extracellular Amyloid-beta (Aβ) and intracellular neurofibrillary tangles (NFTs) are referenced as the disease’s major pathological hallmarks (Chen et al. 2019). Several research studies on the neuropathogenesis mechanism of AD insight neuronal calcium dyshomeostasis, mitochondrial dysfunction, oxidative stress, inflammation, and apoptosis as the principal contributing factors to the disease pathology and pathophysiology (Afewerky et al. 2016, 2019; Yan et al. 2018a; Mahaman et al. 2019). Notably, the root extract of *W. somnifera* has been demonstrated to significantly reduce the level of Aβ-induced reactive oxygen species in N-SH neuron-like cells (Singh and Ramassamy 2017) and reverse cognitive impairments by ameliorating dendritic, axonal, and synaptic integrity in animal models of AD (Uddin et al. 2019). Furthermore, the *W. somnifera* root extracts have shown promising results in the aspects of cognitive and memory improvement in several preclinical studies of AD, including a pilot study in adults with mild-cognitive degeneration (Choudhary et al. 2017a). The protective mechanisms of *W. somnifera* root extract against AD pathology presumably involve binding of the extract–biochemically active constituents to the active motif of Aβ, thereby preclude Aβ fibril formation. However, multidisciplinary extensive preliminary research is needed before disseminating the plant extracts for the AD pharmacological approach.

#### Role of *W. somnifera* against Parkinson’s disease

Parkinson’s disease (PD) is the second most prevalent progressive neurodegenerative disease of ageing after AD, known for causing locomotor deficits (Marino et al. 2020). The main pathological hallmarks of PD include pronounced degeneration of dopaminergic neurons in the midbrain substantia nigra and aggregation of neuronal Lewy Bodies (Marino et al. 2020). Research studies suggest that environmental toxins, such as certain pesticides, that induce mitochondrial dysfunction are the major contributing factors of PD (Wirdefeldt et al. 2011; Bragoszewski et al. 2017; Chen et al. 2017). However, the PD pathogenesis mechanisms remain unclear, and there is no definitive PD-modifying therapy to date. Remarkably, *W. somnifera* extracts have been documented to reverse PD-pathology, including levels of dopamine in the striatum (Manjunath and Muralidhara 2013), and improve locomotor defects in the *Drosophila melanogaster* model of PD (Manjunath and Muralidhara 2015). Further, reported studies in several PD models indicated that *W. somnifera* extracts significantly reverses the Parkinsonian phenotype (Surathi et al. 2016). Although further studies are required to determine the protective mechanisms of *W. somnifera* extract against PD, this plant active constituent’s distinct antioxidant and anti-inflammatory properties likely attribute to correcting mitochondrial aberration and dopamine levels in the striatum.

#### Role of *W. somnifera* against Huntington’s disease

Huntington’s disease (HD) is an autosomal dominant neurodegenerative disorder that causes a progressive loss of locomotor coordination and cognition (Jimenez-Sanchez et al. 2017). An inherited huntingtin gene in chromosome 4 is known to induce nerve cells damage in the HD brain (Nance 2017) through mechanisms that are not well understood. Notably, the root extract of *W. somnifera* has been revealed to ameliorate biochemical parameters, including antioxidant enzymes, responsible for protecting huntingtin protein-induced lesions in basal ganglia tissue and the behavioural outcomes of HD animal models (Kumar and Kumar 2009). Additionally, reported studies of *W. somnifera* extracts pharmacological effect suggest potential roles of the extracts to decrease the lipid-peroxidation and choreiform movements in an animal model of HD (Dar et al. 2015). The GABAergic and antioxidant regulation capability of *W. somnifera* root extract make the plant root extracts suitable neuroprotective candidates against HD; however, research studies are required to elucidate the mechanisms.

### Reproductive impotence

Infertility is regarded as the inability to become pregnant upon regular unprotected intercourse for a year. Infertility could be caused by several factors such as the male factors, a disorder in ovulation, abnormalities in uterine, tubular obstruction, peritoneal factors, or cervical factors, which ultimately emanate from previous reproductive tract infection and complications, exposure to a toxin, among several other factors (Lindsay and Vitrikas 2015). Sexual dysfunction and infertility happen to be critical health concerns worldwide (World Health Organization 2007). The absence of gonadotropin, a hormone in the functions of men and women’s reproductive system stimulated from the pituitary gland, also known as Follicle-Stimulating-Hormone, affects the normal quantitative and qualitative spermatogenesis even in animals (Sharma et al. 2015). Notably, several injectable medications, including gonadotropin-releasing hormones (GnRH), human chorionic gonadotropin (hCG), recombinant follicle-stimulating hormones, and human menopausal gonadotropin (hMG) and the oral medications, such as clomiphene citrate, which are administered for improving abnormal semen, stimulating gonadotropin, and ultimately enhancing spermatogenesis are associated with many shortcomings ranging from high cost to toxicity and various side effects (Buchter et al. 1998). In addressing these reproduction problems, phytomedicine has played a significant role, and a considerable number of natural products have been reported to be efficacious, with positive outcomes (Bussmann and Sharon 2006; Bussmann and Glenn 2010). Besides, properties such as low cost, availability with little to no side effects have given plant resources preference over existing conventional medicine. One plant species that has attracted attention as an aphrodisiac or potent remedy for reproduction impotence is *W. somnifera* (Weiner and Weiner 1994). It contains withanolides—the principal bioactive function of the plant (Walvekar et al. 2013; Nasimi et al. 2018b) and is present in herbal formulations marketed in many countries, especially in Asia (Adhikari et al. 2018). Previous studies reported that *W. somnifera* enhances steroidal hormones and improves sexual distress in both males (Ilayperuma et al. 2002; Kiasalari et al. 2009; Nejatbakhsh et al. 2016; Durg et al. 2018) and females (Dongre et al. 2015). Extracts of *W. somnifera* mildly stimulates the release of gonadotropin hormones in adult rats (Kataria et al. 2015; Rahmati et al. 2016) and improves human menopausal syndrome (Mali et al. 2015). Excessive free radicals, the reactive oxygen and nitrogen species, are generated when the antioxidants level is comparatively low in a body system and produced from the subcellular compartment of testes lead to cell damage and ultimately oxidative stress. Oxidative stress that attacks the fluidity of the sperm plasma membrane and DNA integrity in the sperm nucleus (Agarwal et al. 2003) has been reported to be inhibited by *W. somnifera* (Kulkarni and Dhir 2008; Walvekar et al. 2013) owing to its antioxidant properties. Several studies have validated the aphrodisiac, spermatogenic, and fertility effect of the root, leaf, stem, and fruit extracts of *W. somnifera* both in humans not only in the open literature (Bussmann and Sharon 2006; Mahdi et al. 2009; Ahmad et al. 2010; Tandon and Yadav 2020) but also as patents (Majeed et al. 2019; Gokaraju et al. 2020) and in animals (Abdel-Magied et al. 2001; Kaspate et al. 2015).

*W. somnifera* extracts could be used for the treatment of oligospermia (Dongre et al. 2015) and enhancing libido (Kyathanahalli and Manjunath 2014). In humans, clinical investigations on the efficacy of *W. somnifera* have been reported (Ahmad et al. 2010; Gupta et al. 2013; Dongre et al. 2015; Khalil et al. 2015), including a pilot study on the clinical investigation of the spermatogenic activity of standardized capsule of *W. somnifera* root extract on 46 male patients with oligospermia (< 20 million/mL) focusing on estimating their semen parameters and serum hormone levels after a 12-week treatment. In the study, 225 mg full-spectrum capsule of *W. somnifera* root extract was administered orally-3-times daily for 90 days to 21 patients and compared with 25 patients on placebo. The results demonstrated that the sperm count, semen volume and sperm motility increased by 167% (9.59 ± 4.37 × 10^6^/mL to 25.61 ± 8.6 × 10^6^/mL), 53% (1.74 ± 0.58 mL to 2.76 ± 0.60 mL) and 57% (18.62 ± 6.11% to 29.19 ± 6.31%), respectively, at *P* < 0.0001 on the 90th day. Similarly, a very recent triple-blind randomized clinical study compared the effects of *W. somnifera* with pentoxifylline on sperm parameters of 100 idiopathic infertile male patients for 90 days (Nasimi et al. 2018a). The result demonstrated that *W. somnifera* root extract improved sperm parameters without any adverse effect. The mechanism of action of *W. somnifera* on male infertility patients is by suppressing oxidative stress (Tahvilzadeh et al. 2016).

A study in an animal (Kaspate et al. 2015) investigated the aphrodisiac activity of an authenticated hydroalcoholic extract of dried *W. somnifera* root in tubal ligated female Wistar rat. Different concentrations (100, 200, and 300 mg/kg/day) were gavage administered for 21 days with the reading of the sexual behaviour taken on the 11th and 21st days. The results demonstrated that sexual motivation, hormonal level, and histology of the genital organ of the rats increased at doses of 100, 200, and 300 mg/kg/day when compared with the estrous control. *W. somnifera* has been reported to possess a phytoremedial effect as an antidote against arsenic-induced reproductive toxicity (Sharma et al. 2015). It is also evident as a potent enhancer of sexual function and behaviour by increasing testosterone levels and regulation of NF-κB and Nrf2/HO-1 pathways in male rats (Sahin et al. 2016). However, there exists some variance in the reproductive potency effects of *W. somnifera*. For instance, it was reported to possess spermicidal activity (Nasimi et al. 2018b), decrease sex accessory organs in adult male albino rats (Mali et al. 2015), and have no relief improvement in psychogenic erectile dysfunction when compared with placebo (Mamidi and Thakar 2011). Although Azgomi et al. (2018) surmised that such variations could be as a result of the interventions or protocols adopted in the trials. It is, therefore, crucial and beneficial that adequate clinical evidence is reached on *W. somnifera* either in humans or animals using standard doses before its integration as medicine for the treatment of sexual dysfunction issues.

### Other chronic diseases

#### Anticancer potentials of *W. somnifera*

Various in vitro and in vivo studies have proven the potential efficacy of *W. somnifera* in the prevention and treatment of different types of cancers as a result of its rich pool of pharmacologically relevant secondary metabolites and chemical constituents. Scientific findings have evidenced that two chemical components of *W. somnifera,* namely withanone and withaferin A, could be employed in the development of cancer drugs (Vaishnavi et al. 2012). Further, several studies have pointed out some well-defined mechanisms through which *W. somnifera* exhibits anticancer activities, including regulation of DNA damage pathway, induction of oxidative stress, nuclear factor (NFk-b), signal transducer and activator of transcription-3 (STAT3) signalling, PI3K (phosphoinositide-3-kinase)/AKT (a serine-threonine protein kinase), mitogen-activated protein kinase (MAPK) signalling, and inhibition of angiogenesis (Widodo et al. 2008). Mayola et al. (2011) showed the anticancer effect of withaferin A on melanoma cells through the induction of oxidative stress mechanisms (Mayola et al. 2011). A report by Yang et al. shows that withaferin A in combination with radiation stimulated apoptosis by the generation of reactive oxygen species and several other mechanisms, including the stimulation of MAPK signalling, Bcl-2 downregulation, and caspase-3 activation (Yang et al. 2011). Hahm et al. (2014) showed that cytotoxicity induced by withaferin A is cell line-specific and much dependent on the MAPK signalling pathway (Hahm et al. 2014). Withaferin A has also given a positive result against the formation of the mammosphere in human breast cancer through apoptosis and complex III mitigation (Lee et al. 2012). Withaferin A has also proved efficacious against experimental mammary tumours by inhibiting the expression of vimentin (Lee et al. 2016) and interfering with the cytoskeletal architecture of β-tubulin (Antony et al. 2014). Withaferin A also decreased the tumour size of mammary gland carcinoma in a transgenic mouse (Kim and Singh 2014; Kim et al. 2020). Withaferin A proved positive results in a kidney cancer cell line by eliciting numerous apoptotic pathways and cleavage of Poly (Adenosine diphosphate-ribose) polymerase (PARP) through the inhibition of the STAT3 pathway (Um et al. 2012). Several other studies have recorded the anticancer effects of *W. somnifera* (Setty et al. 2017; Dar et al. 2019; Kim et al. 2020; Maheswari et al. 2020; Saggam et al. 2020).

#### Effect of *W. somnifera* on stress and stress-related obesity

Stress, a normal body reaction, increases concentrations of cortisol in blood that subsequently can lead to several other cascades of events. Interestingly, the architecture of the human body is naturally designed to experience stress and react to it. Stress can be regarded as positive or negative. Many well-documented studies have shown that *W. somnifera* is a notable stress reliever and consequently able to help in weight loss. Particularly studies on root extract of *W. somnifera* have shown significant effect as a potential candidate for lowering stress and stress-induced eating that may, consequently, lead to weight loss. There is linkage pointing to stress as an essential factor in obesity due to its direct effect on eating habits and the rate of metabolism in such a manner that may contribute to weight gain. Researchers have thought that any drug or medicinal plant extract or combination with the potential ability to reduce stress could be a promising candidate for individuals who are susceptible to stress-induced obesity. In line with these existing assumptions on stress as a contributing factor in obesity, *W. somnifera* is one medicinal plant noteworthy for its potentials to significantly reducing some symptoms of anxiety (Pratte et al. 2014). In a recent study conducted by Choudhary et al., obese people with high-stress index fed with 300 mg of root extract of *W. somnifera* two times daily for 8-consecutive weeks showed a significant improvement in some measure indices, including body weight, levels of the stress-related hormones, and reduction in stress-induced eating and feeling (Choudhary et al. 2017b). This result suggests that the root extract of *W. somnifera* can be employed for the management of stress-induced obesity in individuals. Other researchers also reported similar results for the anxiolytic effect of *W. somnifera* (Chandrasekhar et al. 2012; Candelario et al. 2015; Manchanda and Kaur 2017; Lopresti et al. 2019; Salve et al. 2019; Deshpande et al. 2020).

#### *W. somnifera* as an antimicrobial agent

In folkloric medicine, *W. somnifera* has proven efficacious against infections. The antimicrobial activity of *W. somnifera* depends on the organism involved, and this is achieved through several mechanisms, including but not limited to cytotoxicity, immunopotentiation, and gene splicing. To scientifically validate the use of *W. somnifera* in traditional medicine several research studies have reported effective-antifungal and antibacterial effects of the plant extracts in a laboratory setup, including a favourable zone of inhibitory effects on gram-positive *Enterococcus spp*. and *Staphylococcus aureus* (Bisht and Rawat 2014). A similar finding was recorded on the effect of *W. somnifera* against gram-negative bacteria (Singh and Kumar 2011; Alam et al. 2012).

## Commercialization of *W. somnifera* in Africa

*W. somnifera* is a plant widely known for its nutritive value and medicinal uses. It is commonly found and cultivated in India (Durg et al. 2018). Although it does not have a uniform distribution across Africa, it is prevailing in countries including South Africa, Lesotho, Botswana, Eswatini (formerly Swaziland), Namibia, Eritrea, Ethiopia, Sudan, South Sudan, Djibouti, Egypt, Tanzania, Swaziland, Mali, Nigeria, Liberia, and Congo (Burkill 1985; Dzoyem et al. 2013; Gaurav et al. 2015). However, it is usually not found in all provinces of these countries (Gaurav et al. 2015).

*W. somnifera* is majorly seen as a weed in several places, as it usually grows on its own without prior planting. However, it grows and survives best in areas of less rainfall or water, preferably dry and warm heated areas; and as a result, it rarely exists in all regions (Gaurav et al. 2015). This primary growth factor of *W. somnifera* suggests that the drier parts of Africa can give more attention to its deliberate cultivation (Shanmugaratnam et al. 2013). Several studies are available on the cultivation and management of *W. somnifera* (Rajeswara et al. 2012; Shrivastava and Sahu 2013) that enlightens as references for an actionable investment project on the herb and its extracts.

Traditionally, every part of *W. somnifera* has been used in various forms to treat different diseases and ailments like cough, skin infections, malaria, fever, dysentery, asthma, stress, ulcer, infertility, insomnia, etc. (Verma and Kumar 2011; Umadevi et al. 2012; Nasimi et al. 2018b). Due to the enormous nutritional value and medicinal uses of *W. somnifera*, there exists an immense market demand for this plant. Notably, the high demand for this plant and its extracts has led to a rise in its cultivation and production globally, which in turn signifies its commercialization significances both in the local and international markets. Over 7000 tons of *W. somnifera* root production is needed yearly, and India, the largest source of the plant product, produces only about 1500 tons each year (Umadevi et al. 2012). Unfortunately, there are limited articles and publications available on *W. somnifera* commercialization in Africa.

Africa is indigenous to many medicinal plants and natural products. However, there is poor documentation of medicinal plants in Africa, including *W. somnifera*. There is, therefore, a need for a comprehensive compilation of and research on plants used in traditional medicine in different regions of Africa. As much as it is known, at the time of writing this literature review, only very few studies have reported on the occurrence of *W. somnifera* in Africa. For instance, the *W. somnifera* occurrences in Congo, Egypt, and Morocco have been documented (Jana and Charan 2018), but there is no substantial information on the plant ethnomedicinal usage and commercialization features in these areas.

Africa is yet to fully tap into the commercialization of *W. somnifera* on a global scale*.* As of the time of this report, there are little or no reference details accessible on the commercialization of this medicinal plant in Africa. Although this plant is abundantly present in Africa, it is recognized only for its usage and demand in a local context. Undeniably local markets are beneficial to start with for basic economic sustenance in communities of interest. However, Africa needs to consider international commercialization because local level markets are typically crude, informal, and unregulated, while the practices concerning traditions and customs in worldwide settings benefit most the entire population. At the moment, most African plant materials are gotten through wild harvesting and wild crafting (VanWyk 2015). The rise in demand and pressure for medicinal plant materials place urgent considerations for deliberate cultivation and management of plants rather than wild harvesting. Noteworthy, the sustainability of *W. somnifera* in its natural flora is crucial as wild harvesting of plant materials can pose a threat to the human race as the plant might not be able to resist pressure from humans and thereby extinct (Street and Prinsloo 2013). Thus, there is a call for large-scale cultivation, production, and commercialization of *W. somnifera* in Africa as this will enhance the growing economy while also reducing the current high rate of unemployment in most African countries. In addition to the economic benefit, the international commercialization of *W. somnifera* in Africa would also promote mutual relationships in several global aspects between Africa and the world. Africa lags other continents of the world when it comes to the commercialization of medicinal plants. The *W. somnifera* large-scale cultivation and commercialization are vital to initiate with aiming for subsequent promotions of the other related medicinal plants of significance due to the proven recent rises in *W. somnifera* products demand in international pharmacological industries alongside the local people needs.

The acceptance and demand of *W. somnifera* in traditional medicine are evidenced in the high number of citations and publications over time. Less than 2% of the medicinal plants predominantly common in South Africa have been made accessible as refined substances in various forms like drugs, dietary supplements, balm, or pills (VanWyk 2008). Ethiopia is one of the other African countries that takes part in the commercialization of *W. somnifera* at the rate of 3.03 USD per kg (Dzoyem et al. 2013); however, there is limited data on the plant use within Ethiopia or which of the plant parts is at most used traditionally. Unlike developed countries, the lack of commercialization of medicinal plants in African countries can be partly traced to a lack of reliable data and market secrecy (Dzoyem et al. 2013). The world’s interest in *W. somnifera* has placed pressure on its demand and thus creates an opportunity for large-scale cultivation for commercialization. For instance, there is a yearly increase in the market demand of *W. somnifera* in India wherein above 4000 hectares of land is exclusively used for this plant cultivation in Madhya Pradesh, a drier Indian state (Shrivastava and Sahu 2013).

It is germane to note that although *W. somnifera* is distinctly recognized among the African indigenous plants with the highest number of research publications of about 1767 papers, there exist not much data on the plant commercialization by the African community (VanWyk 2015). Even with the vast commercialization of *W. somnifera* going on in other parts of the world, wherein this plant is available in various forms and combinations in the market, such as a paste, powder, pellets, capsules, medicinal raw materials, drugs, supplements, or extracts; except that of South Africa, reference data covering this plant commercialization in the Africa continent remains unavailable.

## Conclusions

The acceptance and demand of *W. somnifera* in traditional medicine to prevent or treat several pathophysiological conditions, including cardiovascular, neurodegenerative, and reproductive impotence, place higher demands for the plant’s deliberate large-scale cultivation, production, and international commercialization in Africa. The commercialization of *W. somnifera* will be beneficial to Africa in several ways, including: -

*Awareness—*the sales of this plant will create more awareness about the potential of the region. This will attract international partners, thereby strengthening international relations.

*Financial and economic independence—*the sales of this plant will strengthen the financial power of the involved people and boost their economic independence-alleviating poverty in the region.

*Reduction of the unemployment rate—*commercialization will involve aspects like advertising, marking, and logistics. This will lead to the recruitment of personnel, thereby reducing the unemployment rate.

## Data Availability

Not applicable.

## Abbreviations

GBIF: Global biodiversity information facility
LD_50_: Mean lethal dose
BW: Body weight
AD: Alzheimer’s disease
PD: Parkinson’s disease
HD: Huntington’s disease
STAT3: Signal transducer and activator of transcription-3
PI3K: Phosphoinositide-3-kinase
MAPK: Mitogen-activated protein kinase
PARP: Poly (adenosine diphosphate-ribose) polymerase
